# Reflecting optics in the diverticular eye of a deep-sea barreleye fish (*Rhynchohyalus natalensis*)

**DOI:** 10.1098/rspb.2013.3223

**Published:** 2014-05-07

**Authors:** J. C. Partridge, R. H. Douglas, N. J. Marshall, W.-S. Chung, T. M. Jordan, H.-J. Wagner

**Affiliations:** 1 School of Biological Sciences, University of Bristol, Woodland Road, Bristol BS8 1UG, UK; 2 Department of Optometry and Visual Science, City University London, Northampton Square, London EC1V 0HB, UK; 3 Queensland Brain Institute, University of Queensland, St Lucia, Brisbane, Queensland 4072, Australia; 4 Anatomisches Institut, Universität Tübingen, Ősterbergstrasse 3, Tübingen 72074, Germany; 5 School of Animal Biology, University of Western Australia, 35 Stirling Highway, Crawley, Perth, Western Australia 6009, Australia

**Keywords:** *Rhynchohyalus natalensis*, vision, mirror optics, deep-sea

## Abstract

We describe the bi-directed eyes of a mesopelagic teleost fish, *Rhynchohyalus natalensis*, that possesses an extensive lateral diverticulum to each tubular eye. Each diverticulum contains a mirror that focuses light from the ventro-lateral visual field. This species can thereby visualize both downwelling sunlight and bioluminescence over a wide field of view. Modelling shows that the mirror is very likely to be capable of producing a bright, well focused image. After *Dolichopteryx longipes*, this is only the second description of an eye in a vertebrate having both reflective and refractive optics. Although superficially similar, the optics of the diverticular eyes of these two species of fish differ in some important respects. Firstly, the reflective crystals in the *D. longipes* mirror are derived from a tapetum within the retinal pigment epithelium, whereas in *R. natalensis* they develop from the choroidal argentea. Secondly, in *D. longipes* the angle of the reflective crystals varies depending on their position within the mirror, forming a Fresnel-type reflector, but in *R. natalensis* the crystals are orientated almost parallel to the mirror's surface and image formation is dependent on the gross morphology of the diverticular mirror. Two remarkably different developmental solutions have thus evolved in these two closely related species of opisthoproctid teleosts to extend the restricted visual field of a tubular eye and provide a well-focused image with reflective optics.

## 1. Introduction

As daylight in the ocean is very directional, several mesopelagic fish have developed upward-facing tubular eyes, the dorsal parts of these each being filled with a large spherical lens that produces a focused image on a well-developed main retina that lines the base of the tube. A more rudimentary accessory retina, which receives only unfocused lateral illumination, coats the medial wall of each tube eye [1–6]. Although most tubular eyes of this type are orientated dorsally, in a few species they are rostrally directed. These latter species are thought, however, to position their bodies in the water column such that the eyes usually point towards the water surface.

High sensitivity, which is the primary prerequisite for the eye of an animal that resides in the low light levels offered by the deep sea, requires a large pupil. Most mesopelagic fish, however, are relatively small, making the possession of a large eye, normally required for an enlarged pupil, problematic. Tubular eyes can therefore be regarded as the central portion of a normal spherical eye that has been laterally reduced [4,7], allowing small animals to have eyes with relatively large pupils. The binocular overlap afforded by such eyes will further increase sensitivity [8] and may also provide a cue for determining object distance [1].

Dorsally directed tubular eyes will maximize sensitivity to downwelling daylight against which animals higher in the water column will cast a silhouette. However, at many times of day, and in deeper water, the dominant source of illumination in the deep sea is not sunlight but bioluminescence [9–14], which may provide illumination or light stimuli from any direction. As tubular eyes have a very restricted visual field (the main retina typically receives illumination from less than 50° directly above the animal [15,16]), animals with such eyes will be unaware of any sort of visual stimulus from the side or below.

At least one species (*Macropinna microstoma*) overcomes the limited visual field of a dorsally directed tubular eye by using extensive eye movements [17]. Other mesopelagic fish enlarge the visual field of their tubular eyes by developing laterally directed light-guiding optical specializations, such as the lens pads of scopelarchids [2,3,6,18] and the optical folds of evermannelids [4]. A few also extend their visual fields by having outpockets of their eyes’ lateral walls that are lined with retina [2–5,19,20]. Ventro-lateral illumination reaches these diverticula through an unpigmented ‘window’, either directly or after reflection from an argentea within the lateral wall of the tube eye.

Tubular eyes are found in several families of deep-sea teleost [5] but extensions of their limited visual fields such as the above are rare. Most of the devices for extending the visual field of tubular eyes lack refractive surfaces and therefore allow only unfocused light perception. Two species of opisthoproctids, however, have evolved extensive diverticula that almost certainly provide well-focused images. *Bathylychnops exilis* has dorsally directed spherical eyes and ventrally directed secondary eyes with scleral lenses [21]. *Dolichopteryx longipes*, on the other hand, has dorsally directed tubular eyes as well as extensive ventro-laterally directed diverticula which, uniquely among vertebrates, produce focused images using Fresnel-type mirrors [22].

Here, we describe the diverticulum of another mesopelagic species of opisthoproctid, *Rhynchohyalus natalensis*, that also uses a mirror to produce a focused image in its diverticular eye. This is only the second vertebrate described to use a mirror in this way and it differs in some important respects from the mirror observed in *D. longipes*. The eye of *R. natalensis* has previously been described [3,19] but these authors studied a post-larval specimen and outlined an ocular structure significantly different from that of the larger animal described here.

## 2. Material and methods

**Figure 1.**
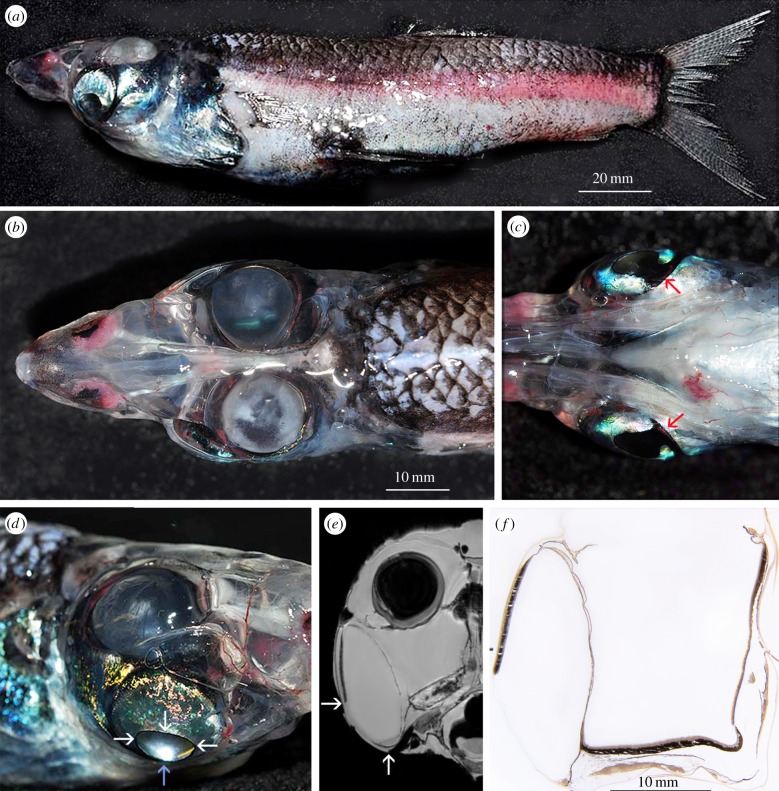
Gross morphology of the eyes of *R. natalensis*. (*a*) Lateral view of specimen shortly after capture; (*b*) dorsal view of head showing the spherical lenses of the dorsally directed tubular eyes; (*c*) ventral view of the head showing the silvery lateral walls and the dark cornea of the diverticulum—the red arrows indicate a medial notch in the diverticular cornea, enlarging the visual field caudo-medially; (*d*) lateral view of the right eye—note the reflection of the flashlight (blue arrow) from the diverticular mirror located inside the eye and observed at the time of collection; (*e*) MRI section of the right half of the head showing the tubular eye including the lens and the lensless diverticulum; (*f*) 25 μm thick resin-embedded histological section of the eye with the lens removed. In (*d*,*e*) the margins of the ventro-laterally facing diverticular cornea are indicated by arrows.

A single *R. natalensis* (standard length 183 mm, figure 1*a*) was caught in the Southern Tasman Sea Abyssal Basin (41°6.2′ S/152°21.8′ E) between 800 and 1000 m depth. It was photographed (figure 1) before fixation in 4% formalin in seawater and subsequent preservation in 70% ethanol.

### (a) Magnetic resonance imaging

The fish was removed from the storage medium and rehydrated by immersion for 2 h in a series of reducing concentrations of ethanol (steps in concentration: 50, 25 and 10%). After rehydration, the fish was placed overnight in 0.1 M phosphate buffer saline (pH 7.4, 300 mOsm kg^−1^) to which was added the magnetic resonance imaging (MRI) contrast agent, 1% ionic Gd-DTPA (Magnevist, Bayer, Germany), prior to MRI following a protocol developed for zebrafish [23]. The sample was placed in an imaging tube containing fomblin oil (perfluoropolyether, Ausimont, Morristown, NJ, USA) to reduce artefacts caused by air–tissue boundaries and to prevent dehydration. The tube was placed in a custom-built surface acoustic wave coil (M2M Imaging, Brisbane, Australia). A 16.4 T magnet was used combined with a 700 MHz wide-bore microimaging MRI system (Bruker Biospin, Karlsruhe, Germany). The fish was imaged at 50 μm isotropic resolution using a T_2_*-weighted three-dimensional FLASH sequence with the following acquisition parameters (modified from the protocol developed for zebrafish; [23]): reception time (TR) and echo time (TE) pulses were 50 and 12 ms, respectively, eight averages. The total imaging time was 14 h. Images were analysed using OsiriX (v. 4.1.2, Pixmeo, Switzerland) image processing software.

### (b) Histology

After MRI examination, the isolated eyes were postfixed in 2.5% glutaraldehyde and 1% osmium tetroxide. After removal of the lenses, the eyes were embedded in Epon, serially sectioned at 25 µm and mounted on plastic slides. Sections were photographed on a Zeiss stereomicroscope and selected sections and areas were re-sectioned at 1 µm or 80 nm. Some of the thick and semi-thin sections were stained with a mixture of methylene blue and Azur II. In order to test the refractive properties of structures, unstained sections were examined in a combination of dark-field and polarized light illumination (see [22] for details). Light and electron micrographs (Zeiss/LEO EM912) were recorded digitally.

### (c) Modelling the geometric optics and image focusing potential of the diverticular eye

A photomicrograph of a midline dorsoventral section of the ocular diverticulum was digitized using ImageJ v. 1.46 64 bit for Mac OSX [24] to delineate tissue layers comprising the sclera, retinal outer limiting membrane (OLM) and the diverticular mirror lateral surface. These digitized data were used to create a Matlab v. R2012b (MathWorks, MA, USA) model of the diverticulum in which the fate of rays entering the eye's ventral cornea was traced in two dimensions (i.e. in the plane of the section). The model's premises included: the diverticulum's function is to focus light from distant point sources onto the OLM; all of the OLM and mirror surface is used in image formation; the axial orientations of the rod outer segments (ROSs) converge at a point outside and lateral to the eye; the eye has a primary axis, rays entering at this angle being focused at the centre of the OLM; and ROSs have an acceptance angle beyond which incident rays are fully rejected. The acceptance angle was calculated to be ±19.95° using ROS and extracellular fluid refractive indices of 1.4106 and 1.335, respectively, from the data of Sidman [25], as used previously in similar exercises by Kaplan [26], and the equations given by Enoch [27]. The mirror's surface topography was modelled in three ways: using the digitized surface data smoothed with an eighth-order polynomial; as a best-fitting arc of a circle; and as a best-fitting parabolic section. As in our previous publication [22], some ocular dimensional parameters, such as ROS axial convergence point and the angles of putative guanine plates in the diverticular mirror, were allowed to iterate to provide a solution that maximized OLM irradiance and minimized defocus of rays originating from given points in space.

### (d) Modelling the physical optics and reflectivity of the crystal stack

The thickness values used for the crystal layers and cytoplasm gaps are summarized in the results and correspond to a disordered ‘chaotic’ stack structure. Using this histological information from the mirror of the ocular diverticulum, it is possible to estimate the spectral, angular and polarization properties of the reflectivity of this class of crystal stack by using the optical transfer matrix methods (developed by Jordan *et al*. [28]) for physically analogous reflectors in fish skin. This method incorporates the high birefringence of biogenic guanine crystals [29,30], which we model as uniaxial with refractive indices of 1.83 perpendicular to the stacking direction and 1.46 parallel to the direction of stacking. The cytoplasm gaps are assumed to have a refractive index of 1.33 [28,30,31]. In order to account for the optical response of the bulk structure, the reflection spectra were ensemble averaged over a set of 1000 random stack configurations [28,31]. Implicit in this approach is the assumption that the average structure is homogeneous throughout the mirror.

## 3. Results

### (a) Gross morphology of the eye

The eye of *R. natalensis*, like that of other opisthoproctids, consists of both a tubular portion and a lateral diverticulum. The dorsally directed tube eyes are most apparent in dorsal view (figure 1*b*), while the cornea of the diverticulum can be seen when viewed from the side or from below (figure 1*c*,*d*). The scleral walls of the eye are lined internally by a choroidal argentea and therefore appear silvery (figure 1*d*). The eye, like that of some other opisthoproctids, lies within a wide, dome-like dermal transparent capsule.

The organization of the extraocular muscles in *Rhynchohyalus* is similar to that in mobile eyes of *M. microstoma* [17] suggesting that *Rhynchohalyus* too may be capable of extensive eye movements (electronic supplementary material, figure S1).

**Figure 2.**
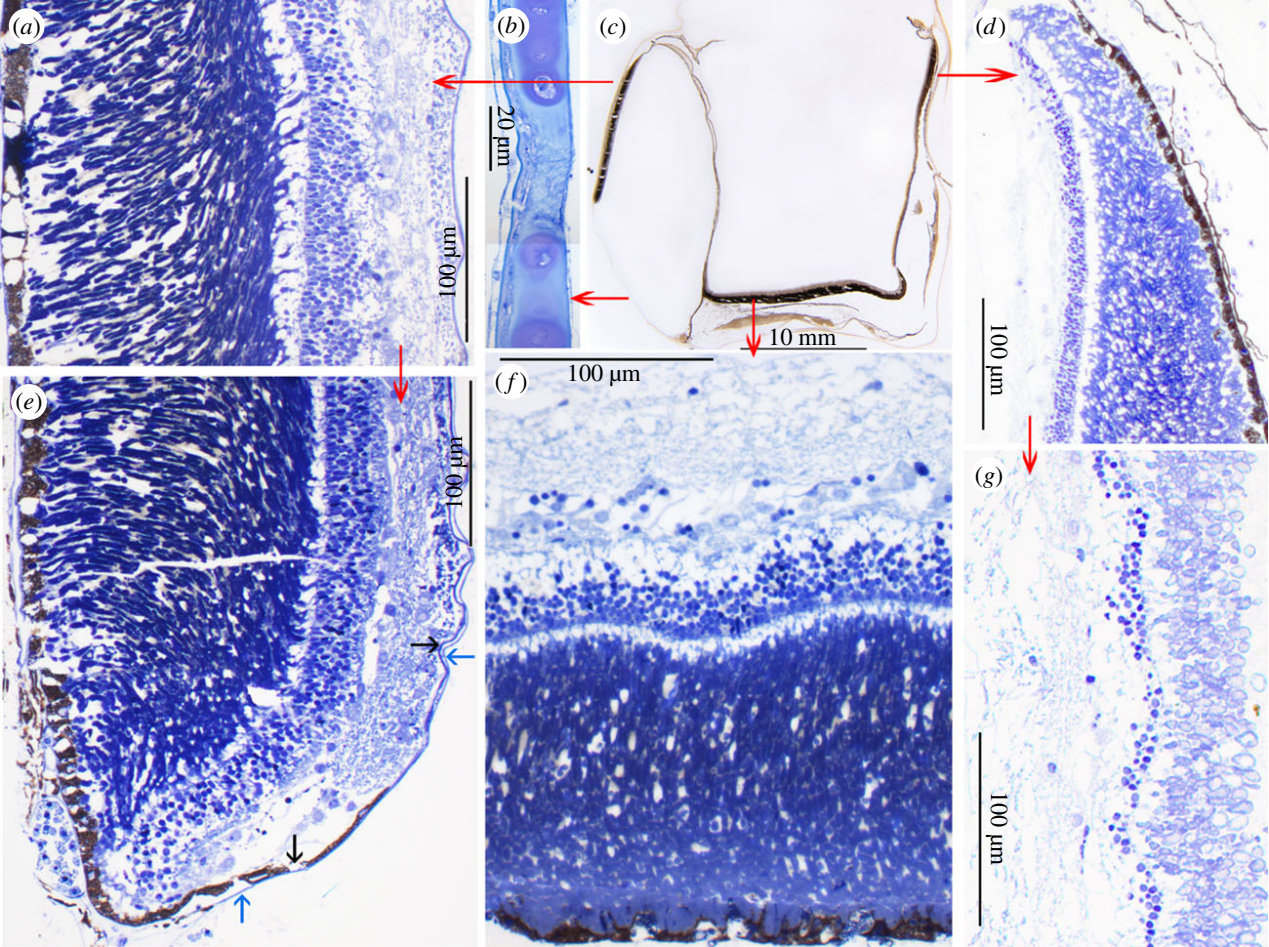
Fine structure of the diverticular cornea and retinae of *R. natalensis*. (*a*) Lateral diverticular retina; (*b*) diverticular cornea; (*c*) 25 μm thick resin-embedded histological section of the entire eye after removal of the tube eye lens; (*d*) dorsal termination of the tube eye medial accessory retina; (*e*) ventral termination of the diverticular retina; note the dorsally directed reflection of the reduced choroid tissue layer, over the photoreceptive retinal surface (indicated by blue arrows) and the similar reflection of the retina, reduced to a simple ciliary epithelium (indicated by black arrows); (*f*) main retina of the tube eye; and (*g*) accessory retina in the medial wall of the tubular eye.

The diverticulum, rostro-lateral to the tube eye, is readily apparent in photographs (figure 1*d*), MRI scans (figure 1*e*; electronic supplementary material, figure S6) and histological section (figure 1*f*). It runs down the entire length of the tubular eye exceeding its ventral margin by several millimetres (maximum height: 23.7 mm; maximum width: 10 mm). The lateral wall of the diverticulum is relatively flat and lined by an argentea, except for an oval transparent area (‘cornea’; maximum diameter: 11.5 mm) facing approximately 45° ventro-laterally. Seen from the ventral side, this cornea has a conspicuous notch medially that would admit light not only from directly ventral but possibly even from the contralateral side (figure 1*c*). Apart from the epithelial outer lining, the transparent cornea is composed of dense fibro-collagenous tissue and/or irregular plates of hyaline cartilage (figure 2*b*).

### (b) Retinal fine structure

The main retina of the *R. natalensis* tube eye is approximately 250 µm thick and includes four layers of rods each between 25 and 30 µm long and about 3 µm in diameter (average: 3.23 ± 0.58 µm s.d., *n* = 20); it has no obvious specialization such as an area of increased photoreceptor density (figure 2*f*). The thin retinal pigment epithelium contains numerous melanosomes and sparsely distributed tapetal crystals. This main retina extends about 2 mm up the medial walls of the tubular eye, where there is a sharp transition to the accessory retina which shows the normal layers of a retina but at a substantially reduced total thickness (100–150 µm) and includes one or two rows of short (15 µm) ROSs (figure 2*g*). Interestingly, towards the dorsal margin of the accessory retina there is a region, about 5 mm wide, where rod thickness and density is increased, with one or two additional rod layers (figure 2*d*). On the lateral side of the tubular eye, and especially lining the septum separating the diverticulum from the main eye, the accessory retina is reduced to a simple ciliary epithelium lacking photoreceptors or other retinal cells. In the diverticulum, the retina, which is little different to that of the main retina in the tube eye, is restricted to the flat lateral wall (figure 2*a*).

### (c) Structure of the medial diverticular mirror

The lateral diverticular retina of *R. natalensis* cannot be illuminated directly (except possibly, and to only a minor extent, via the medial notch in the cornea) and photoreceptors in the diverticular retina can essentially only be illuminated by light reflected from the medial wall of the diverticulum. In *D. longipes*, with a similarly positioned diverticular retina, indirect illumination and a focused image is achieved via a highly reflective medially positioned mirror [22]. It seems likely a similar adaptation is present in *R. natalensis*, which also has a mirror inside the diverticulum eye that was observed in the fresh specimen and can be seen through the cornea (figure 1*d*).

**Figure 3.**
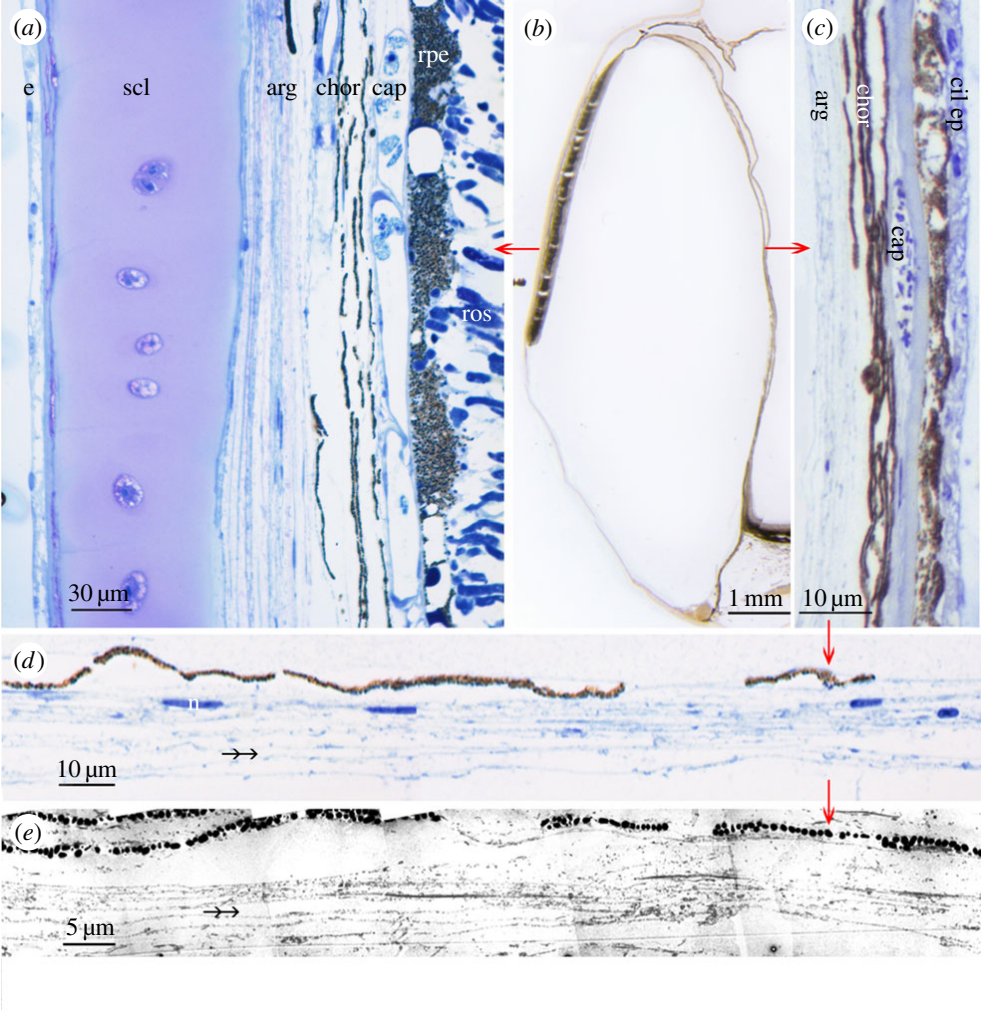
Fine structure of the *R. natalensis* diverticulm. (*a*) Lateral wall of the diverticulm showing the epidermis (e), outer sclera (scl), the choroidal argentea (arg), the pigmented (chor) and vascular (cap) layers of the choroid and the retina including the pigment epithelium (rpe) and rod outer segments (ros); (*b*) 25 μm thick resin-embedded histological section of the entire diverticulum. (*c*) Septum dividing the diverticulum from the main tube eye consisting of a reflective inner layer derived from the lateral argentea (arg), a central layer continuous with the pigmented (chor) and vascular (cap) layers of the lateral choroid and the ciliary epithelium (cil ep) of the accessory retina of the tube eye; (*d*) higher magnification light micrograph of the presumed reflective layer on the surface of the medial diverticular wall; n, nucleus of a fibrocyte or iridocyte; (*e*) electron micrograph of the same layer. The double-headed arrows indicate the ‘ghosts’, i.e. empty intracellular spaces that presumably contained guanine crystals, which have been lost during prolonged storage of the tissue in fixative.

The central component of the septum dividing the tubular portion of the eye from the diverticulum is a choroidally derived layer (see below) containing capillaries of varying diameter, numerous melanocytes and loose fibro-collagenous tissue, lined on both sides by a prominent basal membrane of which the one facing the ciliary epithelium of the tubular eye corresponds to Bruch's membrane (figure 3*c*). Lining the diverticular face of the septum are elongated cells containing three to four layers of thin and empty ‘ghost-like’ spaces. Owing to their similarity to the reflective argentea on the lateral wall of the diverticulum (figure 3*a*), we are confident that the empty spaces correspond to reflective crystals, probably guanine (by reference to other silvery reflective tissues in teleosts), that have dissolved during the long interval between fixation and preparation for histology. A similar effect can be seen by the silvery appearance of the freshly caught specimen disappearing in the preserved specimen. Using dark-field illumination and polarized light, one or two thin lines of residual reflecting particles are observed (electronic supplementary material, figure S2). The crystal ghosts are separated by leaflets of cytoplasm, both of which were measured (see below). Their orientation is always parallel to the basal membrane of the septum (figure 3*c–e*; electronic supplementary material, S5). The space between the presumed guanine crystals and the basal lamina, separating the epithelial structures from the vitreal cavity of the diverticulum, appears artificially swollen with loosely arranged fibrous material and scattered melanosomes.

### (d) Origin of the diverticular mirror

To understand the origin of the reflective crystals in the medial wall of the diverticulum, it is necessary to examine the lateral wall of the diverticulum, which consists of the following well-developed layers (starting internally): the retina, a choroid consisting of an inner vascular layer, a layer of melanocytes and a well-developed argentea, covered externally by a cartilagenous sclera (figure 3*a*). At the ventral margin of the diverticular retina next to the cornea, the diverticular retina ends abruptly (figure 2*e*) and includes a short region resembling the proliferation zone that forms the retinal margin in ‘normal’ hemispheric eyes. At the ventral retinal margin, the retinal pigment epithelium and retina are reduced to a thin bilayered sheet, corresponding to a ciliary epithlium, that wraps around this region and continues dorsally over the surface of the retina. It is accompanied by a thin second layer of fibrocytes and connective tissue corresponding to the choroid. These layers cover the retina proper on its vitreal surface and run dorsally (figure 2*e*; electronic supplementary material, S3).

Further dorsally, the diverticular retina thins and continues as ciliary epithelium (electronic supplementary material, figure S4). The inner epithelia derived from the retina and choroid, however, reflex ventrally and form the inner surface of the diverticular septum. On reaching the septum, the inner choroidal layer once more expresses the argentea, thereby forming the diverticular mirror. Medial to this, choroidally derived melaoncytes and vasculature, together with the ciliary epithelium, form part of the septum separating the diverticulum from the tube eye.

### (e) Modelling of the geometric optics and image focusing of the diverticular eye

**Figure 4.**
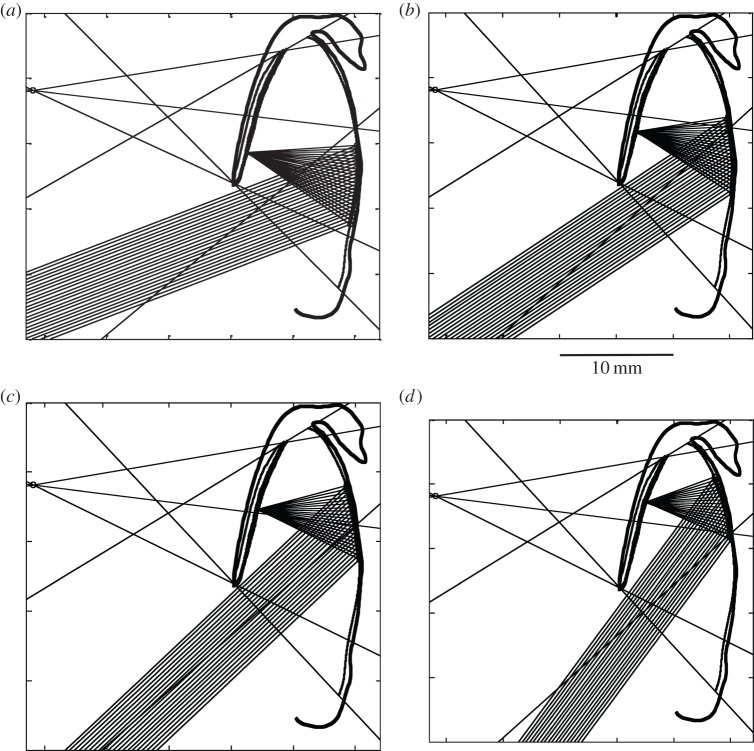
Ray tracing and the performance of the diverticular mirror as a focusing device. Light rays entering the ventral cornea of the diverticular eye from a distant point source are reflected from the lateral surface of the mirror and brought to a focus at the OLM of the diverticular retina. The thickness of the bundle of rays brought to focus is determined by the acceptance angle of the ROSs. In this example of the results of the iterative two-dimensional ray-tracing model, ROS axes diverge from a point (indicated by a small circle at the left of the figures) located some 16 mm lateral to the eye, the primary axis of the eye is 218.3° in the ventro-lateral visual field (indicated by the heavy line entering the eye via the ventral cornea) and mirror plate angles are allowed to vary from the mirror surface tangent. Well-focused images are formed for point sources located at four angles, ranging in steps of 10° from 200 to 230° (inclusive) from the horizontal, shown in subfigures (*a*–*d*), respectively. Reflective plate angles required for this precision of focusing range ±5° about a mean of +5° from the surface tangent, which is less than we are able to resolve from the available tissue samples.

The ray-tracing model was relatively insensitive to the exact mirror surface (polynomial, arc or parabola) considered, but a significant improvement in eye performance was obtained when the angles of the plates of the mirror were allowed to diverge slightly from being exactly parallel to the mirror's surface. A series of tracings, for rays entering from different distant points in the latero-ventral visual field, and in which plate angles in the mirror diverge from surface tangents by ±5° about a mean of +5°, is shown in figure 4.

### (f) Modelling of the physical optics and the reflectivity of the crystal stack

Histological examination of the diverticular mirror showed that, typically, it comprised three to four leaflets of crystals separated by layers of cytoplasm each about 0.11 μm (±0.03 μm s.d., *n* = 25) in thickness, the average thickness of the putative guanine crystals being 0.41 μm (±0.08 μm s.d., *n* = 25), with an average length of 3.27 μm (±1.02 μm s.d., *n* = 25). The thickness of the crystals is considerably greater than is required for an ideal narrowband quarter-wave multilayer ‘stack’ that is tuned to optical wavelengths. This would require a crystal thickness of approximately 0.04–0.09 μm [32]. Instead, the high variation in crystal thickness suggests that the crystal stack could function as a broadband ‘chaotic’ reflector (albeit with a low number of layers). Crystal reflectors of this type are found in the skin of the largehead hairtail *Trichiurus lepturus* and the silver scabbardfish *Lepidopus caudatus* [31] as well as in the iridophores of the common carp *Cyprinus carpio* [33].

**Figure 5.**
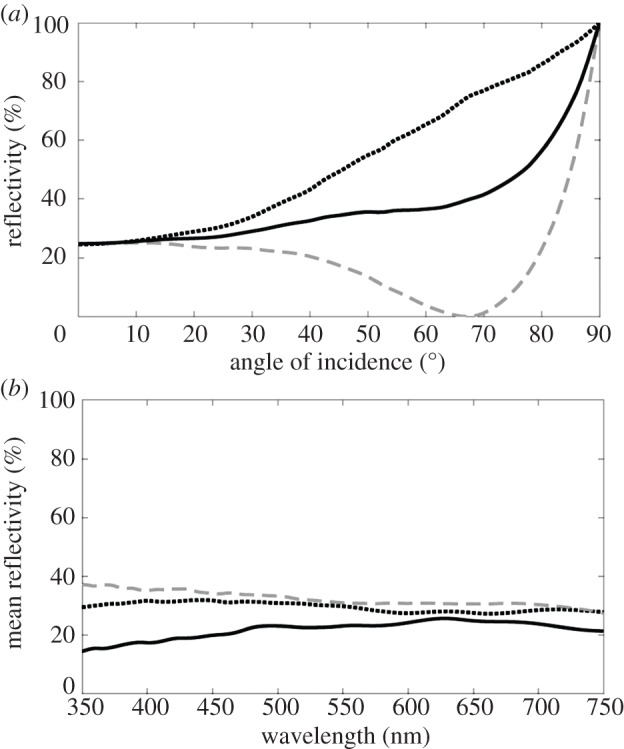
Reflectivity of the diverticular mirror. Angular and polarization dependence of the reflectivity of the diverticular mirror at 475 nm. The dotted black line is for s-polarized light, the dashed light grey line is for p-polarized light and the solid black line is the mean reflectivity averaged over both polarization components. (*b*) Spectral dependence of the mean reflectivity of the diverticular mirror (averaged over both polarization components). The solid dark grey line is for normal incidence, the dotted black line is for 45° and the dashed light grey line is for 60°. The plots (*a*,*b*) illustrate the angular and spectral insensitivity of the mean reflectivity that is predicted from the transfer matrix model of the crystal stack.

Figure 5*a* shows the predicted angular and polarization dependence of the reflectivity of a crystal stack with four crystal layers at a wavelength of 475 nm (which represents ‘blue’ light typical of that in the deep sea, whether from sunlight or bioluminescence [12]). As rod photoreceptors are essentially insensitive to the polarization of light entering them end-on, it is the mean reflectivity (averaged over both polarization components) that is relevant to the information content of the convergent rays. The predicted mean reflectivity is angularly insensitive over the range 0–65°, where it is approximately 30%. Figure 5*b* shows the spectral dependence of the mean reflectivity over wavelengths 350–750 nm. The predicted mean reflectivity is also spectrally insensitive over the range of angles of incidence at which it is angularly insensitive, with values typically in the range 25–35%. The predicted reflectivity spectra in figure 5*b* are similar in bandwidth to ‘chaotic’ fish skin multilayer structures [28,31,33], but with lower absolute reflectivity owing to the low number of layers in the structure. The reflectivity for a crystal stack with three crystal layers produces qualitatively similar angular and spectrally insensitive behaviour, but the reflectivity is lower and typically in the range 20–25%. As has been shown elsewhere [31], the reflectivity that is associated with disordered chaotic reflectors of the type found in *Rhynchohyalus* is relatively insensitive to the exact details of the multilayer stack dimensions. If layer thicknesses disorder were greater than our estimates, broadband reflexion in the visible wavelength regime would still result, with a decrease in percentage reflectivity; if disorder were less, we would see an accompanying decrease in the reflexion bandwidth and an increase in percentage reflectivity. The latter scenario would, however, be unlikely to result in the ideal narrowband reflexion that is associated with quarter-wave stacks as the estimates of the layer thicknesses are far from the required periodicity.

## 4. Discussion

Although some invertebrates use mirrors to form images [34–36], to our knowledge reflective optics have only been described in one vertebrate species [22]. This report is therefore only the second description of a mirror being used to focus light in any vertebrate. It is perhaps surprising that mirrors are not more widely used as image-forming devices in vertebrates as reflective tapeta and argentea are readily available to form the basis of an image-forming reflector. Mirrors would seem to offer some advantages over lenses for forming images particularly because they do not suffer from aberrations to the same degree as thick lenses. In addition, accommodation is relatively easily achieved by small displacement of the mirror away from the retina to focus on closer objects [22], but we lack direct observation of the insertion of the necessary muscles in *R. natalensis*.

There has been a previous description of the *R. natalensis* eye [19] that differs significantly to what we report here. However, the specimen previously examined was a much smaller, post-larval, individual. It possessed a much smaller and simpler diverticulum than the one described here, which was similar to that described in some other mesopelagic fish [2,3,5]. It seems likely that this represents an earlier ontogenetic stage of the larger and more complex adult diverticulum described here.

Although the reflective diverticula of *D. longipes* [22] and *R. natalensis* appear similar, they differ in important respects. In *D. longipes*, the angle of the reflective plates varies considerably with position in the mirror, forming a Fresnel-type reflector in which reflective plates are far from being parallel to the mirror's surface. In *R. natalensis*, however, the gross geometry of the diverticular eye dictates that the reflective plates should lie almost parallel to the mirror's surface for a well-focused image to be obtained, although some small divergence from the surface tangent, unresolvable in our specimen, is predicted by our two-dimensional ray-tracing models. Naturally, three-dimensional ray tracing would provide a more definitive understanding of the focusing potential of the diverticular mirror but this will require access to tissue in better condition, both in terms of gross morphology and in terms of reflective plate histology. In the interim, two-dimensional ray tracing of a midline section of the diverticular eye (a region where we have most confidence of the structure's anatomy) demonstrates that rays in a vertical plane originating from a point source in the latero-ventral visual field can be brought to a good focus. This conclusion, *a priori*, is not a forgone conclusion and shows that the medial wall of the diverticulum is potentially capable of image formation by reflection, based largely on its shape and distance from the retina, with image quality being further enhanced by very small angular departures of the reflective plate angle from being parallel to the mirror's surface. In addition, despite having relatively few crystal layers, the predicted spectral and angular insensitivity of the reflectivity of the disordered crystal stack is suggestive that the structure preserves spatial information when focusing rays upon the ROSs. Most strikingly, in *D. longipes*, the mirror originates from the retinal pigment epithelium, whereas in *R. natalensis* it derives from the choroidal argentea. This major ontogenetic difference suggests differing evolutionary origins of the diverticular reflectors in the two species, despite their close phylogenetic affinity and the ultimately convergent function and adaptive value of the diverticular mirrors.

The apparent complexity and seeming perfection of the conventional vertebrate eye has sometimes been taken as evidence against the very idea of evolution although, in truth, the eye is far from perfect and no more complex than most other organs. In fact, as Darwin himself realized [37], the existence of a variety of eyes with different degrees of complexity, from a simple light-sensitive cell to a fully developed eye, provides one of the best examples of how complex organs might evolve in a surprisingly limited number of generations [38]. Nonetheless, more complex bipartite eyes using both reflective and refractive optics, such as those described here for *R. natalensis* and previously for *D. longipes* [22], remain unusual and require explanation in evolutionary terms. Several members of the Opisthoproctidae have ocular diverticula, ranging from simple small outpockets in *Winteria* sp. and *Opisthoproctus* sp. [2,3,5] to the complex type described here for *R. natalensis* and elsewhere for *D. longipes* [22] or to the scleral lens containing diverticulum of *B. exilis* [21]. This family of teleosts thus presents a highly unusual taxon, exhibiting diverse and unique ocular morphologies that extend the characteristics and capabilities of more common tubular eyes. Further understanding of the value of these adaptations will depend on a combination of detailed anatomical examination and mathematical modelling of ocular performance, combined with knowledge of the group's evolutionary history derived from molecular genetics.

## Notes

All animal handling was performed under the University of Queensland ethics approval no. SNG/080/09/ARC.
